# Socio-demographic determinants of low sexual desire and hypoactive sexual desire disorder: a population-based study in Iran

**DOI:** 10.1186/s12905-020-01097-0

**Published:** 2020-10-14

**Authors:** Zeinab Hamzehgardeshi, Mina Malary, Mahmood Moosazadeh, Soghra Khani, Mehdi Pourasghar, Narges Alianmoghaddam

**Affiliations:** 1 Sexual and Reproductive Health Research Center, Mazandaran University of Medical Sciences, Sari, Iran; 2 Department of Reproductive Health and Midwifery, Mazandaran University of Medical Sciences, Sari, Iran; 3 Student Research Committee, School of Nursing and Midwifery, Shahroud University of Medical Sciences, Haft-e Tir Square, Po Box 7394736147, Shahroud, Iran; 4 Health Sciences Research Center, Faculty of Health, Mazandaran University of Medical Sciences, Sari, Iran; 5 Gastrointestinal Cancer Research Center, Non-communicable Diseases Institute, Mazandaran University of Medical Sciences, Sari, Iran; 6 Research Center of Diabetes, Mazandaran University of Medical Sciences, Sari, Iran; 7 Department of Psychiatry, Psychiatry and Behavioral Sciences Research Center, Addiction Institute, Mazandaran University of Medical Sciences, Sari, Iran; 8 School of Public Health, Massey University, Palmerston North, New Zealand

**Keywords:** Women’s health, Sexual behavior, Sexual dysfunction, Reproductive health, Iran

## Abstract

**Background:**

Various socio-demographic factors have been introduced as the determinants of Low Sexual Desire (LSD), but whether these variables can also contribute to the Hypoactive Sexual Desire Disorder (HSDD), remains uncertain. In this study, we sought to identify the socio-demographic determinants of LSD and HSDD in Iranian women of reproductive age.

**Methods:**

This was a population-based, cross-sectional study of 1000 married Iranian women of reproductive age (16–49 years) who met the inclusion criteria. The participants were chosen using the systematic random sampling method from all the healthcare centres in the city of Sari, Iran. LSD was defined as a score no higher than 33 on the Sexual Interest and Desire Inventory-Female (SIDI-F). The sexually-related personal distress was considered as a score of at least 11.0 on the Female Sexual Distress Scale-Revised (FSDS-R), and HSDD was determined based on the sum of those scores. Descriptive statistics were used to describe the socio-demographic characteristics and a chi-square test was run for data analysis using grouping variables. Multivariate logistic regression test was also employed to adjust the effect of confounding variables.

**Results:**

The mean score of sexual interest/desire among women was 30.6 ± 10.5. After adjusting the effect of confounding variables, logistic regression showed that socio-demographic variables including age at first intercourse, length of marriage and the level of satisfaction with income were significantly associated with both LSD and HSDD (P < 0.01). While advancing age (P < 0.001) and body mass index (P < 0.01) were just predictors of LSD.

**Conclusion:**

Some socio-demographic factors could predict LSD in women, while they were not associated with HSDD. In other words, some factors associated with LSD do not instigate sexually-related personal distress, which is one of the criteria necessary for the diagnosis of HSDD.

## Background

Hypoactive Sexual Desire Disorder (HSDD) is one of the most common types of female sexual dysfunction that can affect women of all ages. It is worth noting that HSDD is different from Low Sexual Desire (LSD) often experienced in daily life [1]. A sexual complaint can only be considered a sexual disorder when the diagnostic and statistical criteria for sexual dysfunctions are met and when that sexual problem has caused personal distress [2].

According to the *Diagnostic and Statistical Manual of Mental Disorders, 4th Edition, Text Revision* (DSM-IV-TR), an LSD which can cause significant personal distress is the primary indicator of HSDD. However, in the *Diagnostic and Statistical Manual of Mental Disorders, 5th Edition* (DSM-5), HSDD is categorized under the Female Sexual Interest/Arousal Disorder (FSIAD), which is a controversial reform. Regardless of the benefits of amendment in diagnostic criteria, decades of research based on the DSM-IV-TR criteria for the diagnosis of HSDD has shaped the basis of our understanding of LSD-related distress’ primary symptoms, epidemiology, and clinical management [3]. Also, arguments about the epidemiology of FSIAD have been based on the extrapolation of studies using HSDD rather than FSIAD criteria [4].

According to a European study of women with LSD, the prevalence of LSD varies from 11 to 53%. However, only 22–65% of those women with LSD reported that experiencing LSD-related distress which is required for the diagnosis of HSDD [5].

Sexual desire is a multi-factorial and multidimensional phenomenon which may differ significantly amongst individuals due to their cultural values that need to be addressed accordingly [6]. For example, HSDD may root in biological, psychological, sexual, and social factors [7]. According to an international study of sexual health, women diagnosed with HSDD reported greater sexual and marital dissatisfaction, hopelessness, frustration, anger, loss of femininity, and low self-esteem compared to those women without HSDD [8].

Various studies have yielded different results regarding the socio-demographic factors affecting LSD and HSDD. Although some of those studies pointed out that the risk of LSD and HSDD increases with age [7, 9, 10], a European study reported that only LSD is associated with ageing [5]. Understanding the role of socio-demographic factors is essential in screening individuals for LSD and HSDD.

So far, very few international studies have been conducted in this field. Although social determinants of sexual satisfaction in Iranian women have been studied [11], no studies have been conducted in Iran to identify the socio-demographic factors associated with LSD and HSDD among reproductive-age married women. Therefore, we sought to investigate the socio-demographic factors associated with LSD and HSDD. It is hoped that raising awareness regarding this issue among Iranian women could be a major step forward in planning future national policies aimed at promoting women’s sexual and reproductive health.

## Methods

### Design and data collection

This population-based, cross-sectional study was conducted with a 2-stage cluster sampling design in Sari, Iran. Firstly, the necessary scientific permissions were attained from Mazandaran University of Medical Sciences. In the second stage, ethical approval for this study was obtained from the Ethics Committee of Mazandaran University of Medical Sciences (IR.MAZUMS.REC.94.1734). Finally, after the collection of research data and data analysis, the results were reported based on the STrengthening the Reporting of OBservational studies in Epidemiology (STROBE) checklist [12].

### Sampling procedure

The 2-stage cluster sampling method was used in this study. For this purpose, all the healthcare centres in the city of Sari were selected to ensure maximum heterogeneity. Then, we used the systematic random sampling method to choose women of reproductive age admitting to those centres. Based on the probability of selection in proportion to population size (or estimated population size), the sample size from each centre was determined. Furthermore, a list of eligible women was prepared and numbered, then a fixed sampling interval was determined. The interval was determined by dividing the population size by the desired sample size. Finally, those women who met the inclusion criteria were contacted and invited to participate in the study.

### Participants

A total of 1000 women of reproductive age recruited from all the women’s health centres in the city of Sari, between October 2015 and January 2016. The research information sheet that was approved by the Human Ethics Committee of Mazandaran University of Medical Sciences shared with the research participants who met the inclusion criteria and agreed to participate in this study. Participants privacy and confidentiality were discussed before obtaining the signed consent form. It is worth mentioning that none of the study participants was under the age of 16, so there was no need for parental or guardian consent. Also, all women with LSD or HSDD were identified and referred to a sexual health professional team for additional assessment and receiving sexual health education, counselling, and/or treatment interventions.

Participants in this study included women of reproductive age who had been married and have been living with their sexual partner for at least 6 months and were willing to participate in the study. Moreover, based on the exclusion criteria of the study, pregnant women and who were breastfeeding during the first six months after birth as well as women with premature ovarian failure were excluded from the study.

### Outcome measurements

First, the socio-demographic characteristics of the participants, including age, age at first intercourse, number of children, length of marriage, Body Mass Index (BMI), level of satisfaction with income, level of education, level of physical activity, current smoking and alcohol use, were recorded. Subsequently, the participants were asked to fill out the Sexual Interest and Desire Inventory-Female (SIDI-F) and Female Sexual Distress Scale-Revised (FSDS-R).

SIDI-F was designed by Clayton et al. in 2006 and consists of 13 items, plus 5 diagnostic items [13]. The probable score for each individual falls within 0 to 51, with higher scores indicating higher sexual desire. The reliability of this tool was proved to be excellent, and the internal consistency of this tool was established with the Cronbach's alpha coefficient of 0.90. The validity of this tool was also examined by correlating it with other valid instruments of sexual function [14]. Moreover, the validity and reliability of the Persian version of this tool have been assessed in Iran. The internal consistency of the Persian version was confirmed with the Cronbach's alpha coefficient of 0.90, and the test–retest method with a two-week interval proved the proper reliability of this tool [15].

FSDS-R was used for the assessment of personal distress, which is key for the diagnosis of HSDD. FSDS-R is comprised of 13 items, rated based on a 5-point Likert scale ranging from 0 (never) to 4 (always), with higher scores (11 and above) indicating greater sexual distress. The original version of the FSDS-R has shown acceptable reliability with the Cronbach’s alpha falling within the range of 0.87 to 0.93 and a high test–retest reliability (with the internal correlation coefficient ranging between 74 and 86%). For determining the reliability of the Persian version of FSDS-R, internal consistency and reliability were assessed using the test–retest method. Furthermore, Cronbach’s alpha coefficient was determined as a measure of internal consistency, while its reliability was assessed using test–retest measurement (repeatability index). The homogeneity of 70% and higher was considered acceptable. Using the test–retest method, internal consistency and reliability were calculated to be > 0.70. Therefore, the Persian version of FSDS-R is considered a valid and reliable tool for assessing distress related to sexual dysfunction in Iranian women and can be used for screening patients with HSDD [16].

Women with LSD (SIDI-F score ≤ 33.0) and sexually-related personal distress (FSDS-R score ≥ 11) were classified as having HSDD. When accompanied by personally-related sexual distress, this score provides a robust definition of HSDD for use in an epidemiologic context.

## Statistical methods

In a study of women aged 20–60 years from 28 cities of Iran, the prevalence of sexual desire problems was found 35% (p = 35%) using the Female Sexual Functioning Index (FSFI) [17]. With an estimated precision of 3% (d = 0.03), a confidence level of 95% (α) (Z = 1.96), and a non-response rate of 10%, the standard sample size was estimated at 1000 women.

Statistical analyses were performed using SPSS, version 18. The normal distribution of the data was confirmed. Then mean and SD values were obtained using descriptive statistics. The socio-demographic factors associated with LSD and HSDD were identified using the chi-square test. A *P*-value of lower than 0.20 in the chi-square test was adopted as representing the critical level for the selection of variables. Those variables with a *P*-value greater than 0.20 were maintained as adjusting factors in the multivariate logistic regression analysis [18].

Multivariate regression analysis was conducted to analyze the relationship between the socio-demographic variables as well as HSDD and LSD. *P*-value of less than 0.05 was considered statistically significant.

## Results

A total of 1000 married women of reproductive age (16 to 49 years with the mean age of 32.09 ± 7.33 years) participated in this study. The socio-demographic characteristics and their correlation with LSD and HSDD are shown in Table 1. All of the socio-demographic characteristics were significantly associated with LSD and HSDD, except the alcohol consumption and physical activity which they were only associated with LSD.

**Table 1:** Socio-demographic characteristics of study participants & chi-square test results for predictors of LSD & HSDD among women of reproductive age

Variablesand categories	Total	LSD			HSDD		
	Frequency (%)	Frequency	Percentage (%)	P/value	Frequency	Percentage (%)	P/value
*Age (years)*							
< 30	409 (40.9)	163	39.9	P < 0.001	64	15.6	0.03
30–35	276 (27.6)	149	54.0		55	19.9	
36–40	164 (16.4)	115	70.1		40	24.4	
40 <	151 (15.1)	116	76.8		37	24.5	
*Age at first intercourse*							
9–17	180 (18.0)	111	61.7	0.002	55	30.6	P < 0.001
18–20	291 (29.1)	172	59.1		61	21.0	
21 ≤	529 (52.9)	260	49.1		80	15.1	
*Education level*							
Primary-illiterate	25 (2.5)	15	60.0	P < 0.001	4	16.0	P < 0.001
Secondary	391 (39.1)	249	61.9		105	26.9	
University	584 (58.4)	286	49.0		87	14.9	
*Duration of marriage*							
< 2	76 (7.6)	19	25.0	P < 0.001	7	9.2	P < 0.001
2–5	256 (25.6)	100	39.1		25	9.8	
6–10	253 (25.3)	130	51.4		58	22.9	
10 <	415 (41.5)	294	70.8		106	25.5	
*Body Mass Index (BMI)*							
< 25	466 (46.6)	213	45.7	P < 0.001	81	17.4	0.17
25–30	374 (37.4)	215	57.7		77	20.6	
≥ 30	160 (16.0)	115	71.9		38	23.8	
*Number of children*							
None	288 (28.8)	102	35.4	P < 0.001	41	14.2	P < 0.001
One	348 (34.8)	192	55.2		66	19.0	
Two or more	364 (36.4)	249	68.4		89	24.5	
*Satisfaction of income level*							
Never	154 (15.4)	100	64.9	P < 0.001	43	27.9	P < 0.001
Low	217 (21.7)	145	66.8		62	28.6	
Moderate	558 (55.8)	269	48.2		86	15.4	
Much	71 (7.1)	29	40.8		5	7.0	
*Physical activity*							
Never	583 (58.3)	340	58.3	0.008	125	21.4	0.04
Rarely	303 (30.3)	151	49.8		45	14.9	
Almost always	114 (11.4)	52	45.6		26	22.8	
*Current smoking*							
Yes (at least one cigarette in the last month)	29 (2.9)	14	48.3	0.01	9	31.0	0.11
No (never)	971 (97.1)	529	54.5		187	19.3	
*Alcohol consumption*							
Yes (at least once in the last month)	62 (6.2)	18	29.0	P < 0.001	10	16.1	0.4
No (never)	938 (93.8)	529	56.0		186	19.8	

The results of logistic regression regarding the association between the socio-demographic variables and LSD and HSDD are presented in Table 2. As demonstrated, our results suggest that the odds of LSD and HSDD are 50–60% lower in the women with age at first intercourse ≥ 21 ([OR 0.56; 95% CI 0.3–0.9] and [OR 0.4; 95% CI 0.2–0.7], respectively) than the women with first intercourse between 9and 17 years of age.

**Table 2:** Odds ratios from multivariate logistic regression examining factors associated with LSD & HSDD in women of reproductive age

Variables & categories	LSD		HSDD	
	Odds ratio (95% CI)	P/value	Odds ratio (95% CI)	P/value
Age (years)				
< 30	1.0 (ref.)		1.0 (ref.)	
30–35	0.083	1.4 (0.9–2.0)	0.57	1.1 (0.7–1.9)
36–40	0.005	2.1 (1.2–3.7)	0.48	1.2 (0.6–2.4)
40<	< 0.0001	3.1 (1.6–5.8)	0.35	1.3 (0.6–2.7)
Age at first intercourse				
9–17	1.0 (ref.)		1.0 (ref.)	
18–20	0.39	0.81 (0.5–1.3)	0.01	0.5 (0.3–0.9)
21≤	0.01	0.56 (0.3–0.9)	0.002	0.4 (0.2–0.7)
Education level				
Primary	1.0 (ref.)		1.0 (ref.)	
Secondary	0.10	2.1 (0.8–5.4)	0.06	3.0 (0.9–9.6)
University	0.11	2.1 (0.8–5.3)	0.24	2.0 (0.6–6.5)
Duration of marriage				
< 2	1.0 (ref.)		1.0 (ref.)	
2–5	0.10	1.6 (0.8–3.0)	0.88	1.0 (0.4–2.6)
6–10	0.09	1.7 (0.9–3.5)	0.02	3.0 (1.1–8.0)
10 <	0.02	2.4 (1.1–5.4)	0.03	3.2 (1.0–9.6)
Body Mass Index (BMI)				
< 25	1.0 (ref.)		1.0 (ref.)	
25–30	0.36	1.1 (0.8–1.5)	0.78	1.0 (0.7–1.5)
≥ 30	0.006	1.8 (1.1–2.8)	0.89	0.9 (0.5–1.5)
Number of children				
None	1.0 (ref.)		1.0 (ref.)	
One	0.18	1.3 (0.8–1.9)	0.08	0.6 (0.3–1.0)
Two or more	0.45	1.2 (0.7–2.0)	0.06	0.5 (0.2–1.0)
Satisfaction of income level				
Never	1.0 (ref.)		1.0 (ref.)	
Low	0.65	1.1 (0.6–1.7)	0.72	1.0 (0.6–1.7)
Moderate	0.002	0.5 (0.3–0.7)	0.007	0.5 (0.3–0.8)
Much	0.01	0.4 (0.2–0.8)	0.001	0.1 (0.06–0.5)
Physical activity				
Never	1.0 (ref.)		1.0 (ref.)	
Rarely	0.07	0.7 (0.5–1.0)	0.11	0.7 (0.4–1.0)
Almost always	0.02	0.5 (0.3–0.9)	0.50	1.1 (0.7–2.0)
Current smoking				
No (never)	1.0 (ref.)		1.0 (ref.)	
Yes (at least one cigarette in the last month)	0.96	0.9 (0.3–2.4)	0.22	1.7 (0.7–4.2)
Alcohol consumption				
No (never)	1.0 (ref.)		–	
Yes (at least once in the last month)	0.01	0.4 (0.2–0.8)	–	

The odds of LSD were 3 and 2 times higher among women aged 35–40 years (OR 2.1; 95% CI 1.2–3.7) and those over 40 years of age (OR 3.1; 95% CI 1.6–5.8) than women under 30 years of age. However, age was not a factor associated with HSDD.

Moreover, the women with BMI ≥ 30 were at 1.8-fold greater risk of having LSD compared with the women with BMI > 25 (OR 1.8; 95% CI 1.1–2.8). However, BMI also was not related to HSDD.

The odds of reporting LSD and HSDD were approximately 2.4 and 3.2 times higher in the women who had been married for 10 years or longer compared with the women who had been married for less than 2 years ([OR 2.4; 95% CI 1.1–5.4] and [OR 3.2; 95% CI 1.0–9.6], respectively).

The women who expressed moderate and high levels of satisfaction with income showed lower odds of LSD ([OR 0.5; 95% CI 0.3–0.7] and [OR 0.4; 95% CI 0.2–0.8], respectively) and HSDD ([OR 0.5; 95% CI 0.3–0.8] and [OR 0.1; 95% CI 0.06–0.5], respectively).

Among personal habits, alcohol consumption and high level of physical activity were associated with lower levels of LSD ([OR 0.4; 95% CI 0.2–0.8] and [OR 0.5; 95% CI 0.3–0.9], respectively). However, those variables were not associated with HSDD. Regarding alcohol consumption, the World Health Organization (WHO) has stated that the alcohol consumption rate is low in most Muslim countries including Iran [19]. In a systematic review by Chegeni et al. [20], alcohol consumption among the women who participated in the study was reported to vary from 0.2% to 21%. Consistently, in the present study, only about 6% of the participants mentioned alcohol consumption (at least once in the last month). Moreover, there may be some possible limitation in this current study. For instance, the research participants were recruited from a particular city of Iran, Sari, which precludes the generalization of the findings to the whole female population of Iran.

Among the meaningful factors affecting LSD, age < 40 (P < 0.001), BMI (P < 0.01), and satisfaction with income level (P < 0.05) showed the most significant relationships. However, the most important factors affecting HSDD were age at first intercourse, satisfaction with income level, and length of marriage (P < 0.05), which can indicate the importance of socio-cultural factors in the intensity of HSDD.

## Discussion

The present study aimed to determine the socio-demographic factors associated with LSD and HSDD. According to existing research, FSIAD can be best described as a lack of sexual thoughts or fantasies, absence of responsive desire, and diminished or absent feelings of sexual desire or interest. Therefore, FSIAD is linked with an abnormally low or absent sexual drive or lack of incentive to become sexually aroused [21, 22].

Women with HSDD suffer from emotional and psychological problems due to this disorder. The majority of them complain about feeling less feminine, with a major body-image impact, feelings of concern, unhappiness, frustration, anger and even shame as well as being concerned because they were “letting their partner down” [23].

When LSD is accompanied by personal distress, it is clinically known as HSDD [24]. Therefore, it seems that HSDD is a more severe form of LSD and it may require more extensive clinical therapeutic interventions, while LSD can probably be improved with counselling interventions. Therefore, preventive measures are necessary to avoid progression of LSD to HSDD.

Among the factors explored, the socio-demographic variables associated with LSD were age, length of marriage, age at first intercourse, alcohol consumption, level of physical activity, BMI, and level of satisfaction with income. Whereas satisfaction with income, years spent in marriage, and age at first intercourse were the only variables associated with HSDD.

Our findings support the significant role of ageing in the occurrence of LSD (not HSDD). Results of the logistic regression indicated that by controlling other demographic variables, risk of LSD increased with advancing age in women, but does not have any significant association with HSDD. Such a correlation was also observed in the study by Hayes et al. [5] performed in four European countries (i.e., UK, Germany, France, and Italy) and the USA to examine the relationship between age and HSDD. Hayes's findings showed that the risk of LSD tends to increase with advancing age from 11 to 53% in European women and 22 to 32% in American women, but the prevalence of HSDD did not change significantly with advancing age. In other words, the results of that study, similar to our findings, suggested that although increasing age promotes the risk of LSD, the level of distress does not increase with age. Thus, it can be concluded that advancing age does not lead to an increased risk of HSDD, resulting in a relatively constant prevalence of HSDD over time. This finding was also noted in a literature review study on female sexual dysfunction highlighting HSDD [3]. A more recent study (2017) also showed that although age was identified as one of the factors contributing to LSD, it was not reported among factors associated with sexually-related personal distress and HSDD [25]. In a qualitative study in Iran [26], most of the participants were reluctant to have sex even if they had to be sexually active for the sake of their partners’ satisfaction. Some women considered sexual relationships specific to the period of younger age. In other words, asexuality was a culturally accepted practice for older Iranian women. This is why most of the middle-aged women with LSD in this current study did not report sexually-related distress.

Despite the above findings, some studies suggest that there is no relationship between sexual desire and interest and age [27, 28]. Some studies have even shown that sexual desire increases with age [29]. The difference in results can be attributed to the differences in the statistical populations, sample sizes, participants’ age range, and the tools used to assess HSDD. Previous research in this area did not consider sexual distress as an essential criterion for defining HSDD. It seems that women’s age might be one of those important factors that should be taken into consideration when screening for sexual desire problems.

Another factor related to LSD (not HSDD) in this study was BMI. The present study indicated that by controlling other variables, increased BMI elevated the risk of LSD (the odds ratio for LSD in women with BMI ≥ 30 was 1.8 times greater than those with BMI > 25), but it did not result in an increased risk of HSDD. Studies also suggest that there are inconsistent findings on the association between sexual desire and BMI, and they found no difference in the association between LSD and BMI and HSDD and BMI. The results of a study conducted in Turkey showed that BMI was not a risk factor for sexual dysfunction, and no difference was found between healthy weight and obese individuals in terms of sexual desire [30]. Esposito in the study of obesity and sexual dysfunction pointed out that among the sexual activity parameters, sexual desire and pain were not related to BMI, while an inverse relationship was found between weight and the other sexual domains [31].Similarly, Mozaffari et al. in a case–control study of body weight and female sexual dysfunction in Iran reported the same findings [32]. Bajos also reported no difference in sexual dysfunction (i.e., lack of libido, sexual arousal, and sexual pain) between obese and normal-weight women. The relationship between obesity and sexual function is complex. Therefore, it is necessary to address the direct effects of obesity, pathophysiologic comorbidities and the obesity-related psychological factors to prevent LSD and HSDD in women. This inconsistency in findings might be due to the fact that these studies were conducted in different socio-cultural contexts using different tools and sample sizes.

Regarding the length of marriage, the present study found that by adjusting other variables, as the length of time spent in marriage increased, the risk of LSD and HSDD also elevated. Therefore, after 10 years or more of marriage, the possibility of having LSD and HSDD in women would be 2.4 and 3.2 times higher than women who have been married for less than two years. Likewise, the findings of the present study, many other studies have confirmed the inverse relationship between the length of marriage and sexual desire problems. The results of another study conducted among 356 women aged 20–70 years indicated that women who had been in relationships lasting for 20–29 years experienced higher levels of disaffection compared to women who had been in a relationship for less than five years [33]. Pfaus's review also signified that after controlling for the age variable, women's sexual desire had a significant inverse relationship with marriage duration [34]. Kim (2013) also reported that women with HSDD who had been in long-term relationships tended to have lower scores for sexual desire [35]. Therefore, these findings show the necessity of adopting preventive interventions to promote couples′ sexual health throughout their married life.

In this nationally representative sample, age at first intercourse was also identified as a factor associated with both LSD and HSDD, such that having the first sexual intercourse at a very young age can be considered a risk factor for LSD and HSDD. According to the results of Abdo et al. [7] in Brazil, women whose age at first intercourse was more than or equal to 21 were 1.5 times more likely to experience HSDD compared to women whose first intercourse was between 9 and 17 years of age. On the other hand, in a study by Safarinejad et al. [16] in Iran, it was found that a younger age at marriage significantly contributed to sexual dysfunction. The discrepant findings might be due to the different socio-cultural factors in the design of those research studies. In the Western world, women usually experience their first intercourse at a very young age, but in the Eastern world and most of the Muslim countries including Iran, any extramarital sexual relationship is not accepted socially, culturally and legally.

Controlling of the other socio-demographic variables revealed that increased satisfaction with monthly income decreased the possibility of having LSD and HSDD. Therefore, satisfaction with income acts as a protective factor against developing HSDD. It appears that satisfaction with income level lowers the risk of LSD and HSDD through decreasing tensions and stress in women’s lives. This finding is in line with the results obtained by Ghanbarzadeh et al. [36], which highlighted the role of financial problems as a major risk factor for developing LSD in women. Results obtained from another study also revealed that the emergence of sexual desire problems in the face of financial dependency is highly probable [16]. In a study conducted among 1000 married women aged 16–49 years in Egypt, it was demonstrated that most of the participants believed that LSD is associated with socio-economic conditions such as economic stressors and low income [37]. In contrast, a study conducted in China showed that by controlling variables such as age, level of education, and age at marriage, increased average monthly income in women resulted in an improved sexual relationship [38]. It can only be justified by the fact that financial dependence on the spouse causes false interest in couples to have intercourse.

It is noteworthy that despite the non-significant *P*-value for the level of education variable (secondary category), a high odds ratio was observed for this variable (OR 3; 95% CI 0.9–9.6). This result could be due to the limited number of cases with HSDD in the primary-illiterate category (4 cases). Therefore, the level of education should not be disregarded while assessing and managing HSDD.

The strengths of this current study include its sample size of 1000 married women of reproductive age. Also, since sexually related distress is an essential factor in identifying HSDD, in this study SIDI-F and FSDS-R were used for the diagnosis of HSDD. However, this study had some limitations that need to be addressed. As we know, the etiology of LSD or HSDD is complex and multifactorial. Thus, we could not consider all the factors affecting sexual desire such as personal circumstances (e.g., psychosexual, physical, and biological factors) and relational conditions [38]. It is worth noting that the sexual response cycle is a concept of psychophysiological alteration, and any attempt to stage the female sex response, although useful, is artificial [40]. Also, the other possible comorbidities such as having difficulty in getting aroused or lubricated, having difficulty in reaching orgasm, feeling pain during or after intercourse, and experiencing vaginal dryness should be addressed while assessing female sexual desire problems, which were not measured in the study.

Likewise, Kim et al. [35] reported a significant association between sexual desire and other domains of female sexual function, indicating the need to examine other sexual disorders in the evaluation of sexual desire because components of women’s sex response often overlap with each other, such that sexual desire interacts with and partially overlaps with mental arousal [24]. Basson [41] also proposed that the onset of responsive desire is accompanied by sexual arousal to some extent, which shows that sexual desire and sexual arousal are probably a unitary concept, as in DSM-5 they are categorized under FSIAD [3].

Moreover, in this study, the role of the emotional connection between the research participants and their husbands which is an essential aspect in determining female sexual function was not measured. It seems questions such as the stability of the relationship, satisfaction and happiness as well as the sexual and general health of the partner can be used to identify people with LSD and subsequent sexual distress.

In line with this statement, many studies have shown that male partners’ sexual dysfunctions, especially erectile dysfunction and premature ejaculation harm female sexual desire [42–45]. Therefore, it is important to note that clinicians should evaluate the sexual function of both partners, which encompasses several dimensions and needs an interdisciplinary approach. Therefore, further studies need to be done to gain a deeper understanding of socio-cultural contexts of women’s sexual and reproductive health.

## Conclusions

According to the findings of this current study, some socio-demographic factors including women’s age at first sexual intercourse, length of marriage and household income are associated with both LSD and HSDD in married women. This study also highlights the importance of the socio-cultural contexts of women’s sexual interest and desire. We hope that our findings raise women’s sexual health awareness to increase the chance of early detection and holistic intervention including women-centred counselling and therapy in the early stages of sexual dysfunction.

## Data Availability

The raw data produced in this study will not share publicly due to ethical issues and confidential data protection.

## Abbreviations

LSD: Low sexual desire
HSDD: Hypoactive sexual desire disorder
DSM-IV-TR: *Diagnostic and Statistical Manual of Mental Disorders, 4**th** Edition, Text Revision*
DSM-5: *Diagnostic and Statistical Manual of Mental Disorders, 5**th** Edition*
BMI: Body Mass Index
FSIAD: Female sexual interest/arousal disorder
SIDI-F: Sexual interest and desire inventory-female
FSDS-R: Female sexual distress scale-revised
STROBE: Strengthening the reporting of observational studies in epidemiology
